# Supplementary material for: Interaction of Cerium Oxide Nanoparticles and Ionic Cerium with Duckweed (Lemna minor L.): Uptake, Distribution, and Phytotoxicity
Source: Nanomaterials (Basel). 2023 Sep 8;13(18):2523. doi: 10.3390/nano13182523 (PMC10535116; doi:10.3390/nano13182523)
Supplement: Supplementary file 1 [file nanomaterials-13-02523-s001.zip › nanomaterials-2552213-supplementary.pdf]

Supporting information

Interaction of cerium oxide nanoparticles and ionic cerium with duckweed (Lemna minor. L): Uptake, distribution and phytotoxicity

Yang Liu[1], Xuepeng Zhao[1], Yuhui Ma[2, *], Wanqin Dai[2], Zhuda Song[2], Yun Wang[2], Jiaqi Shen[2], Xiao He[2], Fang Yang[1,*], and Zhiyong Zhang[2, 3, *]

[1] Hebei Provincial Key Lab of Green Chemical Technology & High Efficient Energy Saving, School of Chemical Engineering and Technology, Hebei University of Technology, Tianjin 300130, China.

[2] Key Laboratory for Biomedical Effects of Nanomaterials and Nanosafety, Institute of High Energy Physics, Chinese Academy of Sciences, Beijing 100049, China.

[3] School of Nuclear Science and Technology, University of the Chinese Academy of Sciences, Beijing 100049, China.

*** Corresponding author.**

E-mail address: mayh@ihep。ac.cn; yangfang@hebut.edu.cn; zhangzhy@ihep.ac.cn;.

Tel: +86-10-88233215; Fax: +86-10-88235294.

1. Growth related parameters

After exposure, 20 mg fresh frond samples were homogenized in 80% (v/v) acetone, refrigerated at -20°C for 24 hours to extract the photosynthetic pigments. After centrifugation, the absorbance of the supernatant was read at 662, 645 and 470 nm, and chlorophyll a/b, total chlorophyll, and total carotenoid contents were calculated.

On day 0 and day 3, duckweed in each Petri dish was photographed and the leaf areas were measured using ImageJ software. The average specific growth rate (r) was calculated from equation 1.

$$r = \frac{\ln(\mathrm{Area_{final}}) - \ln(\mathrm{Area}_{initial})}{Days} \quad (1)$$

$\mathrm{Area_{initial}}$： Leaf area of each dish at the start of the test;

$\mathrm{Area_{final}}$： Leaf area of each dish at the end of the test;

Days： Number of days from the beginning to the end of the test period

2. Cell death

50 mg of fresh fronds were incubated in 0.025% (w/v) Eb solution for 15 h, and then excess and unbound dyes were thoroughly washed with deionized water. Thereafter, the samples were immersed in ethanol and placed in a hot water bath at 95 °C for 15 minutes to remove the pigment, and then observed under an optical microscope. The excised root tips were treated in the same way as for fronds, except that the incubation time with Eb solution was 3 h. For quantitative assessment, the colored compound was extracted using a mixture of 1% (w/v) sodium dodecyl sulphate (SDS) and 50% (v/v) methanol (the amount of extractant was proportional to the sample

mass) at 50°C for 15 min, and the absorbance of the colored compound was measured at 600 nm.

3. Results

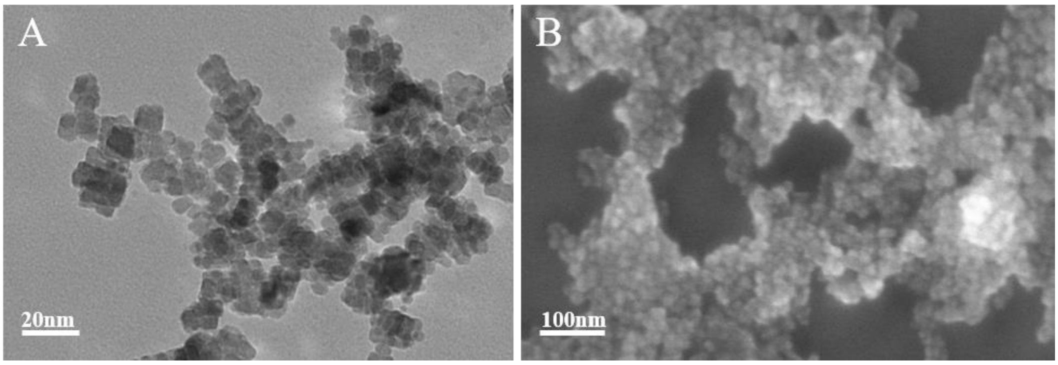

Figure S1. TEM (A) and SEM (B) images of CeO_2 NPs.

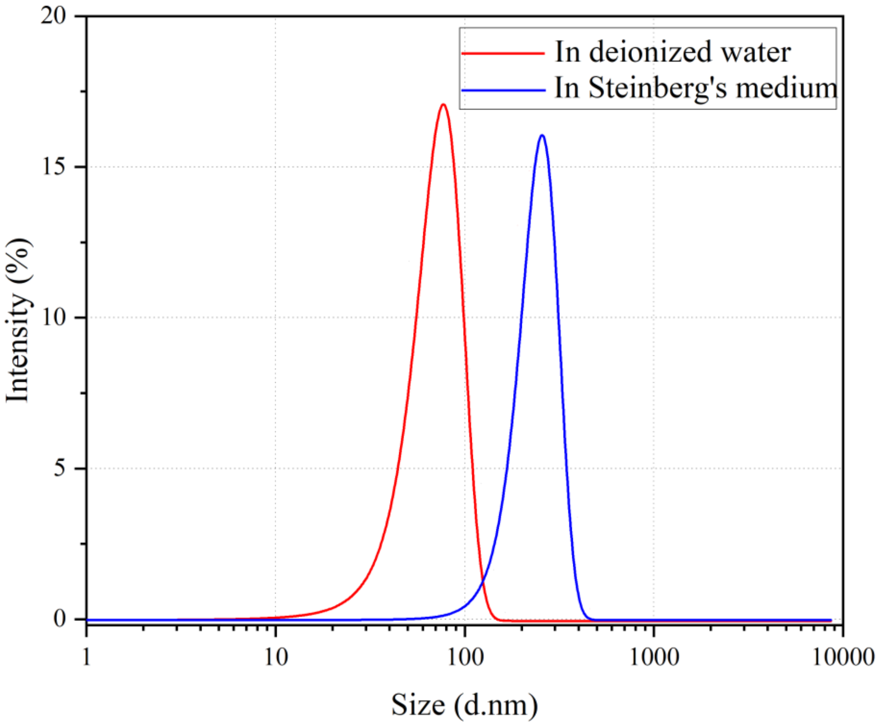

Figure S2. Size distribution of CeO_2 NPs (10 mg/L) in deionized water and Steinberg's medium at 0 day by DLS.

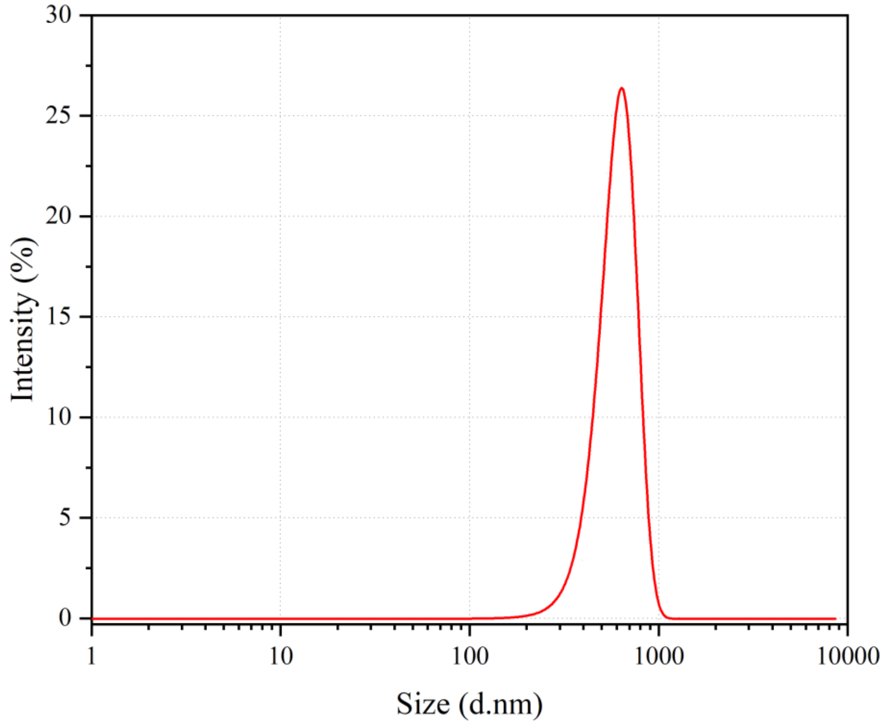

Figure S3. Size distribution of CeO_2 NPs in Steinberg’s medium after exposure.

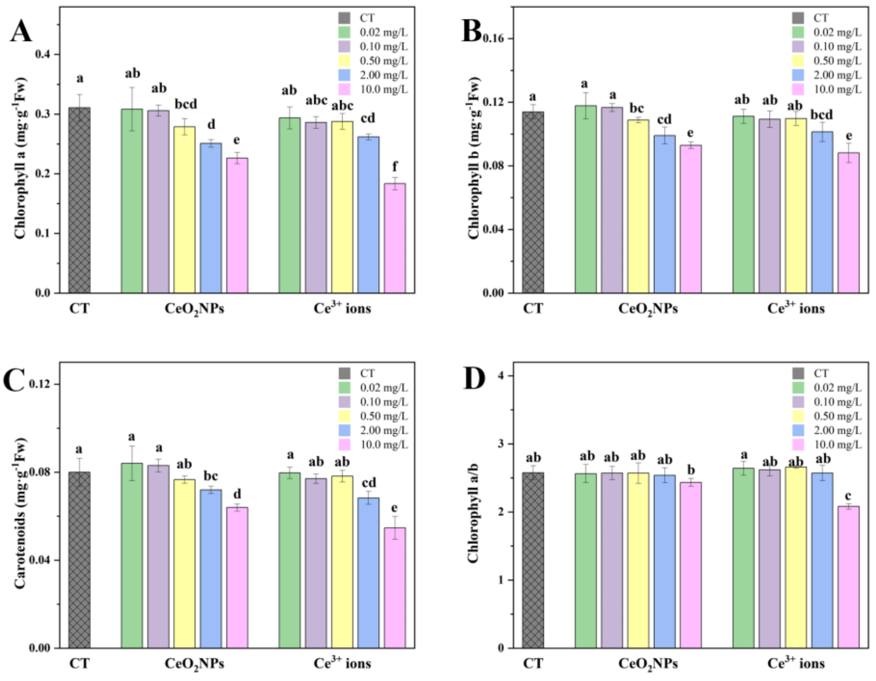

Figure S4. Photosynthetic pigment contents of duckweed under different treatments. A: chlorophyll a; B: chlorophyll b; C: carotenoids; D: chlorophyll a/b. Different letters show significant difference ($p<0.05$).

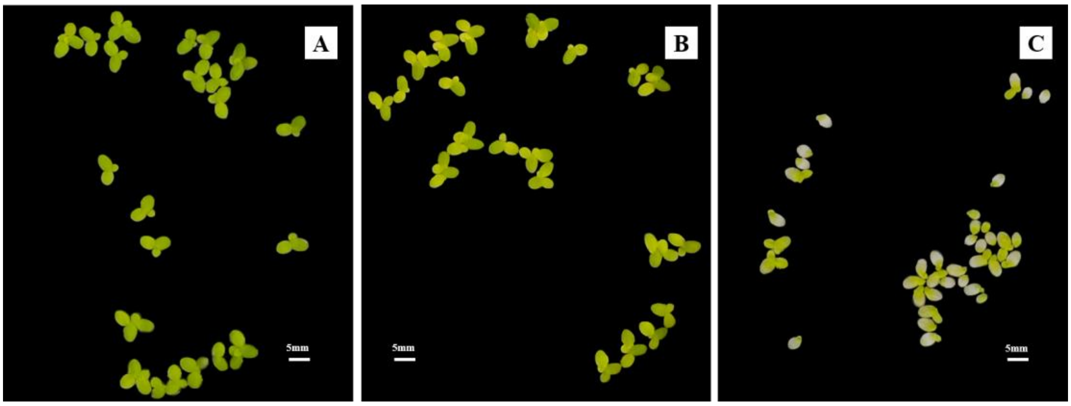

Figure S5. The photographs of duckweed in the control (A), CeO_2 NPs (B), and Ce^{3+} ions (C) groups.

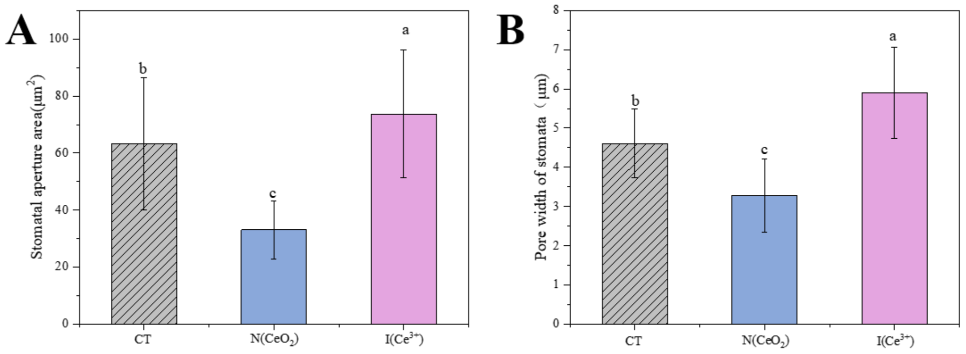

Figure S6. The aperture width (A)and area (B) of stomata on fronds of different treatment groups. Different letters show significant difference ($p < 0.05$).

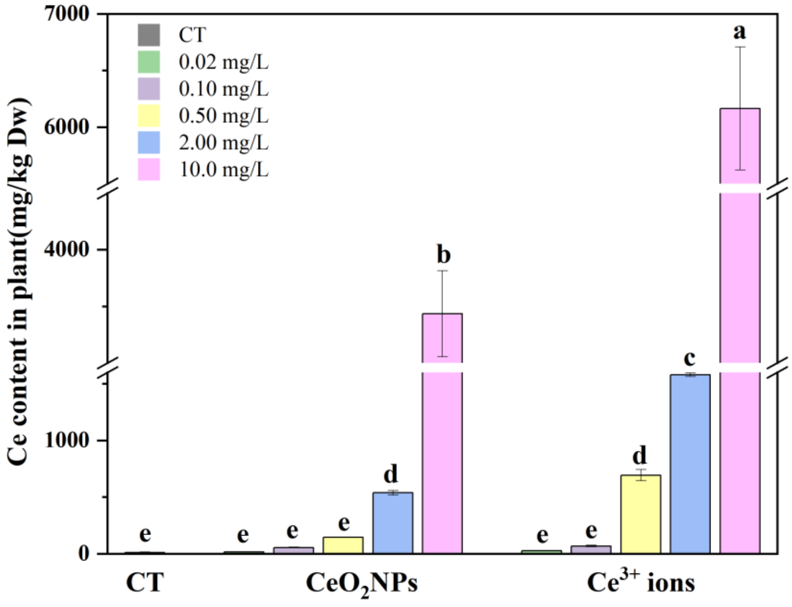

Figure S7. Ce contents in duckweed under different treatments. Different letters show significant difference ($p < 0.05$).

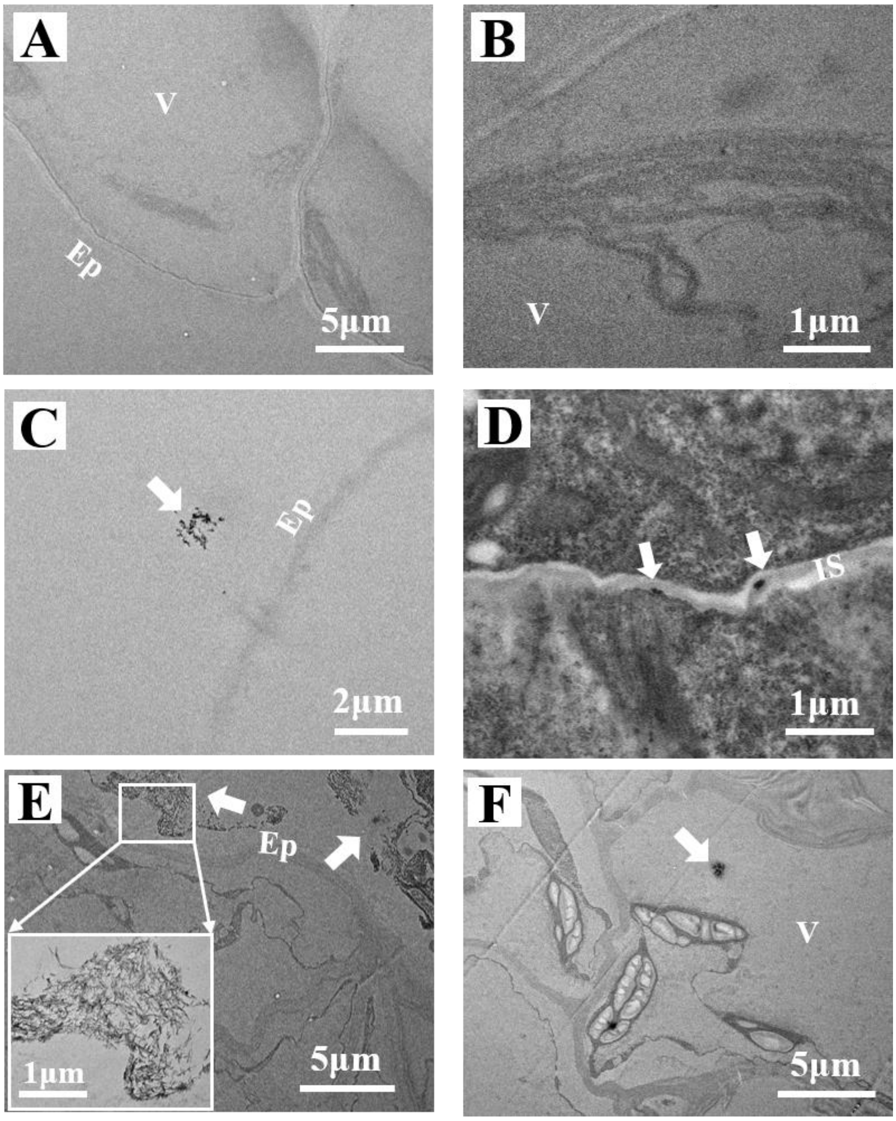

Figure S8. TEM images of root sections of duckweed unexposed (A, B), exposed to 10 mg/L CeO_2 NPs (C, D), and exposed to 10 mg/L to Ce^{3+} ions (E, F). The white arrows indicate CeO_2 NPs or Ce-containing deposits. The inner panel of figure E is enlarged from the rectangle area.

Table S1. BAF of Ce in duckweed after exposure to CeO_2 NPs and Ce^{3+} ions.

Exposure concentration (mg/L)	Log BAF	
	CeO_2 NPs	Ce^{3+} ions
0.02	2.79 ± 0.02	3.10 ± 0.03
0.1	2.72 ± 0.03	2.82 ± 0.05

0.5	2.45 ± 0.01	3.14 ± 0.03
2	2.43 ± 0.02	2.89 ± 0.01
10	2.54 ± 0.05	2.79 ± 0.04

$$BAF\ (L\ kg^{-1}) = \frac{C_{\mathrm{biotic}}}{C_{\mathrm{w}}}$$
